# The mechanism of acquired resistance to irreversible EGFR tyrosine kinase inhibitor-afatinib in lung adenocarcinoma patients

**DOI:** 10.18632/oncotarget.7189

**Published:** 2016-02-04

**Authors:** Shang-Gin Wu, Yi-Nan Liu, Meng-Feng Tsai, Yih-Leong Chang, Chong-Jen Yu, Pan-Chyr Yang, James Chih-Hsin Yang, Yueh-Feng Wen, Jin-Yuan Shih

**Affiliations:** ^1^ Department of Internal Medicine, National Taiwan University Hospital Yun-Lin Branch, Yun-Lin, Taiwan; ^2^ Graduate Institute of Clinical Medicine, College of Medicine, National Taiwan University, Taipei, Taiwan; ^3^ Department of Internal Medicine, National Taiwan University Hospital, and College of Medicine, National Taiwan University, Taipei, Taiwan; ^4^ Department of Molecular Biotechnology, Da-Yeh University, Chang-Hua, Taiwan; ^5^ Department of Pathology, National Taiwan University Hospital, College of Medicine, National Taiwan University, Taipei, Taiwan; ^6^ Department of Oncology, National Taiwan University Hospital, and Graduate Institute of Oncology, Cancer Research Center, National Taiwan University, Taipei, Taiwan; ^7^ Department of Internal Medicine, National Taiwan University Hospital Hsinchu Branch, Hsinchu, Taiwan

**Keywords:** lung adenocarcinoma, afatinib, T790M, acquired resistance, EGFR TKI

## Abstract

**Introduction:**

Epidermal growth factor receptor (EGFR) tyrosine kinase inhibitors (TKIs) are associated with favorable response in *EGFR* mutant lung cancer. Acquired resistance to reversible EGFR TKIs remains a significant barrier, and acquired *EGFR* T790M-mutation is the major mechanism. Second-generation irreversible EGFR TKI, afatinib, had also been approved for treating *EGFR* mutant lung cancer patients, but the mechanism of acquired resistance to afatinib has not been well studied.

**Results:**

Forty-two patients had tissue specimens taken after acquiring resistance to afatinib. The sensitizing *EGFR* mutation were all consistent between pre- and post-afatinib tissues. Twenty patients (47.6%) had acquired T790M mutation. T790M rate was not different between first-generation EGFR TKI-naïve patients (50%) and first-generation EGFR TKI-treated patients (46.4%) (*p* = 0.827). No clinical characteristics or *EGFR* mutation types were associated with the development of acquired T790M. No other second-site *EGFR* mutations were detected. There were no small cell or squamous cell lung cancer transformation. Other genetic mutations were not identified in *PIK3CA*, *BRAF*, *HER2*, *KRAS*, *NRAS*, *MEK1*, *AKT2*, *LKB1* and *JAK2*.

**Methods:**

Afatinib-prescription record of our department of pharmacy from January 2007 and December 2014 was retrieved. We investigated patients with tissue specimens available after acquiring resistance to afatinib. Enrolled patients should have partial response or durable stable disease of treatment response to afatinib. Various mechanisms of acquired resistance to first-generation EGFR TKIs were evaluated. Histology and cytology were reviewed. *EGFR*, *PIK3CA*, *BRAF*, *HER2*, *KRAS*, *NRAS*, *MEK1*, *AKT2*, *LKB1* and *JAK2* genetic alterations were evaluated by sequencing. Statistical analysis was performed using Chi-square test and Kaplan-Meier method.

**Conclusions:**

T790M was detected in half of the lung adenocarcinoma after acquiring resistance to afatinib. T790M is still the major acquired resistance mechanism. First-generation EGFR TKI exposure did not influence the prevalence of T790M in lung cancer acquired resistance to afatinib.

## INTRODUCTION

Lung cancer is a leading cause of cancer-related mortality worldwide [1]. Use of epidermal growth factor receptor tyrosine kinase inhibitors (EGFR TKI) produces dramatic response and favorable prognosis in patients with lung adenocarcinoma harboring epidermal growth factor receptor (*EGFR*) mutations, especially exon 19 deletion and exon 21 L858R point mutations [2]. First-generation EGFR TKIs consisted of gefitinib and erlotinib, which reversibly bind to EGFR and block EGFR signaling. Several phase III trials had proven the effect and benefit of the use gefitinib and erlotinib as first-line agents [3–5].

Second-generation EGFR TKI, afatinib, is an irreversible EGFR TKI which has more potent EGFR inhibition and targets other ErbB-family members. According to LUX-Lung 3 and LUX-Lung 6 studies, afatinib had a significant better response rate and prolonged progression-free survival (PFS) as compared with pemetrexed plus cisplatin or gemcitabine plus cisplatin in patients with treatment-naïve advanced lung adenocarcinoma harboring activating *EGFR* mutations [6, 7]. Afatinib as first-line treatment even prolongs overall survival in patients with exon 19 deletion [8].

However, patients with *EGFR* mutant lung cancer develop disease progression after a median of 10 to 14 months on EGFR TKI. Different mechanisms of acquired resistance to first-generation EGFR TKIs had been reported [9, 10]. Acquired T790M was the major mechanism of acquired resistance to first-generation EGFR TKIs, and it accounts for about a half of the cases with acquired resistance to gefitinib or erlotinib. Several third-generation EGFR TKIs, which irreversibly block T790M mutant *EGFR*, have shown to be effective in patients with acquired *EGFR* mutant lung cancer patients who acquired T790M after treatment failure with previous EGFR TKIs [11, 12]. In addition, other acquired resistance mechanism has been reported; including the development of small cell lung cancer or squamous cell transformation, second point mutations (D761Y or L747S), *MET* amplification, acquired *PIK3CA* or *BRAF* mutation, and epithelial-to-mesenchymal transition [9, 10, 13–16].

Although a preclinical study showed that afatinib could inhibit *EGFR* T790M and block the growth of non-small cell lung cancer (NSCLC) cell lines harboring T790M mutations [17], the clinical trial did not show the overall survival benefit in patients after failure of platinum doublet and first-generation EGFR TKIs [18]. The emergence of acquired resistance remains a significant barrier for afatinib-treated patient in clinical practice. There was only one case report that showed the detection of acquired T790M in lung cancer cells after the development of resistance to afatinib [19]. However, the prevalence of T790M in lung cancer patients with acquired resistance to afatinib has not been studied. *In vitro*, fibroblast growth factor receptor 1 (*FGFR1*) activation was reported as an escape mechanism in human lung cancer cells resistant to afatinib [20]. The other gene mutations and histological evolution of lung cancers after acquiring resistance to afatinib remained poorly understood.

In this study, we explore the prevalence of different acquired resistance mechanisms in tissue specimens taken from patients with acquired resistance to afatinib.

## RESULTS

### Patient collection

**Figure 1:**
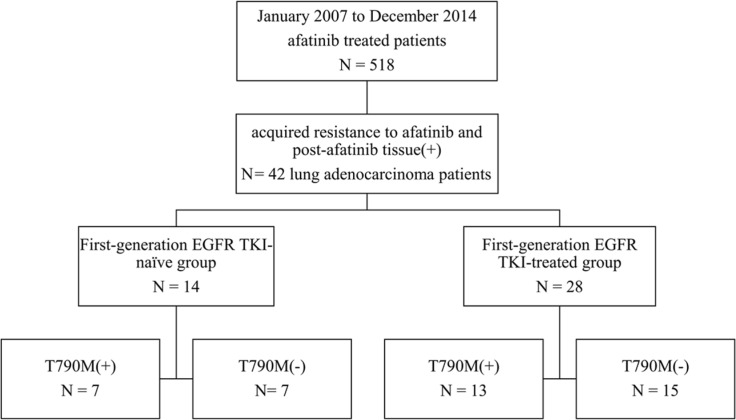
Patient collection flow chart

From January 2007 to December 2014, there were 518 patients who had taken afatinib according to the afatinib-prescription record from the department of pharmacy, NTUH. Those whose lung cancer harboring TKI-sensitive mutations with partial response or durable stable disease (PFS ≥ 6 months) under afatinib treatment and having post-afatinib tissue specimen collected for analysis were enrolled. We enrolled 42 patients in this study (Figure 1).

Of the 42 patients, there were 25 females (59.5%) and 33 never smokers (78.6%). All of them had *EGFR* mutation reports before afatinib treatments, including: 14 deletions in exon-19, 22 L858R and 6 other *EGFR* mutations (L861Q, D770_N711 dupSVD, G719S + S768I, G719C + S768I, L858R + E709G, L858R + S768I). Treatment responses of afatinib were 37 partial response and 5 stable disease (Table 1). Fourteen patients were first-generation EGFR TKI-naïve patients. Twenty-eight patients belonged to first-generation EGFR TKI-treated group, and they received prior first-generation EGFR TKIs treatment before taking afatinib, including: 5 gefitinib, 9 erlotinib, and 14 gefitinib and erlotinib.

**Table 1:** Clinical characteristics of lung adenocarcinoma patients with acquired resistance to afatinib

		All patients	(%)
Total No.		42	(100)
Age, median years		57.5	
(range)		(35.2–81.1)	
Sex			
	Female	25	(59.5)
	Male	17	(40.5)
Smoking			
	Never-smokers	33	(78.6)
	Smokers	9	(21.4)
EGFR mutation			
	Del-19	14	(33.3)
	L858R	22	(52.4)
	Other	6	(14.3)
Pre-afatinib			
	TKI-naïve	14	(33.3)
	TKI using	28	(66.7)
	Gefitinib	5	
	Erlotinib	9	
	Gefitinib and Erlotinib	14	
Line of afatinib			
	1	7	(16.7)
	2	7	(16.7)
	3	3	(7.1)
	≥ 4	25	(59.5)
Afatinib response			
	PR	37	(88.1)
	SD	5	(11.9)

EGFR: epidermal growth factor receptor, Del-19: deletion in exon 19, TKI: tyrosine kinase inhibitor, PR: partial response, SD: stable disease.

The specimens of acquired resistance came from different sites, and the majority was lung tissue via computed tomography or echo-guided biopsy or malignant pleural effusions (MPEs) via thoracentesis. The details of rebiopsy sites and procedure were described in Table 2.

**Table 2:** Details of rebiopsy sites, tissue specimens and the prevalence of T790M

Site	Procedure	Specimen No.	T790M (+)	Prevalence
Right chest wall	echo-guided biopsy	1	0	0%
Mediastinum LN	VATS lymphadenectomy	1	0	0%
Right hip skin	Excisional biopsy	1	0	0%
CSF	lumbar puncture	2	0	0%
MPE	Thoracentesis	18	8	44.4%
Lung	6 CT-guided biopsy 7 Echo-guided biopsy 3 Bronchoscopic brushing 1 Bronchoscopic biopsy 1 VATS lobectomy 1 autopsy	19	12	63.2%

LN: lymph node, CSF: cerebrospinal fluid, MPE: malignant pleural effusion, CT: computer tomography, VATS: video-assisted thoracic surgery.

### T790M prevalence of acquired resistance to afatinib in lung adenocarcinoma patients

The specimens with acquired resistance to afatinib all showed the same sensitizing *EGFR* mutations as the paired treatment-naïve or pre-afatinib treatments tissue specimens. We detected a second-site T790M-*EGFR* mutation in 20 (47.6%) of the 42 specimens with acquired resistance to afatinib, including: 8 MPEs (44.4%) and 12 lung tissues (63.2%) (Table 2). We did not detect other secondary substitutions or point mutation of *EGFR*, including D761Y, L747S and C797S.

Of the 20 patients with acquired T790M, there were 7 from 14 (50.0%) first-generation EGFR TKI-naïve group and 13 from 28 (46.4%) first-generation EGFR TKI-treated group (*p* = 0.827). The sensitizing *EGFR* mutation types of the 20 tumors with acquired T790M included 9 deletion in exon-19 (64.3%; 9 of 14), 10 L858R (45.5%; 10 of 22) and one L861Q (16.7%; 1 of 6) (*p* = 0.142). The clinical factors, including age, smoking, sex, afatinib treatment response, prior first-generation EGFR TKI use were not associated with the detection of T790M after acquired resistance to afatinib (Table 3).

**Table 3:** Comparison of clinical characteristics between patients with acquired T790M and those without T790M

	All patients	Acquired T790M (+)	Acquired T790M (−)	*p* value
**Total No.**	42	20 (47.6%)		22 (52.4%)		
**Age, median years**	57.5	58.2		54.5		0.314^a^
(range)	(35.2–81.1)	(35.9–81.1)		(35.2–78.8)		
**Sex**						0.952
Female	25	12	(48.0%)	13	(52.0%)	
Male	17	8	(47.1%)	9	(52.9%)	
**Smoking**						0.714^*^
Never-smokers	33	15	(45.5%)	18	(54.5%)	
Smokers	9	5	(55.6%)	4	(44.4%)	
**Pre-afatinib**						0.827
TKI-naïve	14	7	(50.0%)	7	(50.0%)	
Prior TKI exposure	28	13	(46.4%)	15	(53.6%)	
***EGFR* mutation**						0.142
Del-19	14	9	(64.3%)	5	(35.7%)	
L858R	22	10	(45.5%)	12	(54.5%)	
Others	6	1^#^	(16.7%)	5	(83.3%)	
**Afatinib response**						0.175^*^
PR	37	16	(43.2%)	21	(56.8%)	
SD	5	4	(80/0%)	1	(20.0%)	

# L861Q + T790M.

* By Fisher exact test.

a By Mann-Whitney *U* test.

EGFR: epidermal growth factor receptor, Del-19: deletion in exon 19, TKI: tyrosine kinase inhibitor, PR: partial response, SD: stable disease.

### Other genetic mutation after acquired resistance to afatinib

The afatinib resistant specimens were examined for histological transformation or genetic mutations. All specimens with acquired resistance to afatinib showed adenocarcinoma. There were no small cell lung cancer or squamous cell transformations.

Because of the limited amount of available specimens, we cannot analyze all possible genes in all samples. The sample numbers for gene mutation analysis were 26 for *PIK3CA*, 25 for *HER2*, 26 for *BRAF*, 26 for *KRAS*, 24 for *NRAS*, 26 for *MEK1*, 24 for *AKT2*, 20 for *JAK2* and 18 for *LKB1*. We did not identify any genetic alternation (0%) in these genes.

### Progression-free survival and post-progression survival of afatinib

**Figure 2:**
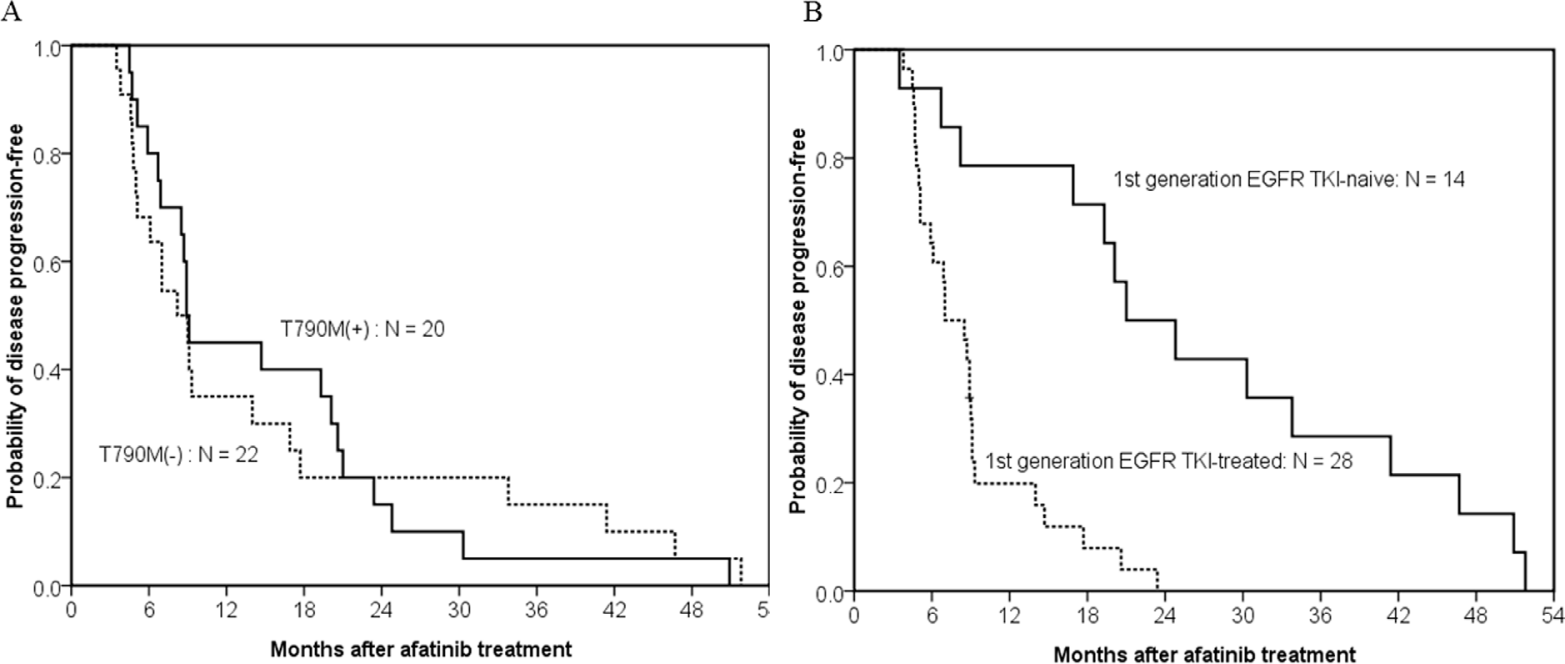
Kaplan–meier curve of afatinib progression-free survival in patients with acquired resistance to afatinib (**A**) The difference in progression-free survival of afatinib treatment between patients with (solid line, *n* = 20) and without acquired T790M-mutations (dashed line, *n* = 22) did not reach statistically significant (median, 8.9 months vs. 8.2 months; *p* = 0.938, by the log-rank test). (**B**) The difference in progression-free survival of afatinib treatment between first-generation EGFR TKI-naïve (solid line, *n* = 14) and first-generation EGFR TKI-treated patients (dash line, *n* = 28) reached statistically significant (median, 21.0 months vs. 7.0 months; *p* < 0.001, by the log-rank test).

Of the 42 patients with acquired resistance to afatinib, there was no difference in PFS following afatinib treatment between patients with and without acquired T790M-mutations (median, 8.9 months vs. 8.2 months; *p* = 0.938) (Figure 2A). First-generation EGFR TKI exposure had influence on PFS of afatinib. The difference in PFS of afatinib reached a statistical significance between 14 first-generation EGFR TKI-naïve and 28 first-generation EGFR TKI-treated patients (median, 21.0 months vs. 7.0 months; *p* < 0.001) (Figure 2B).

**Figure 3:**
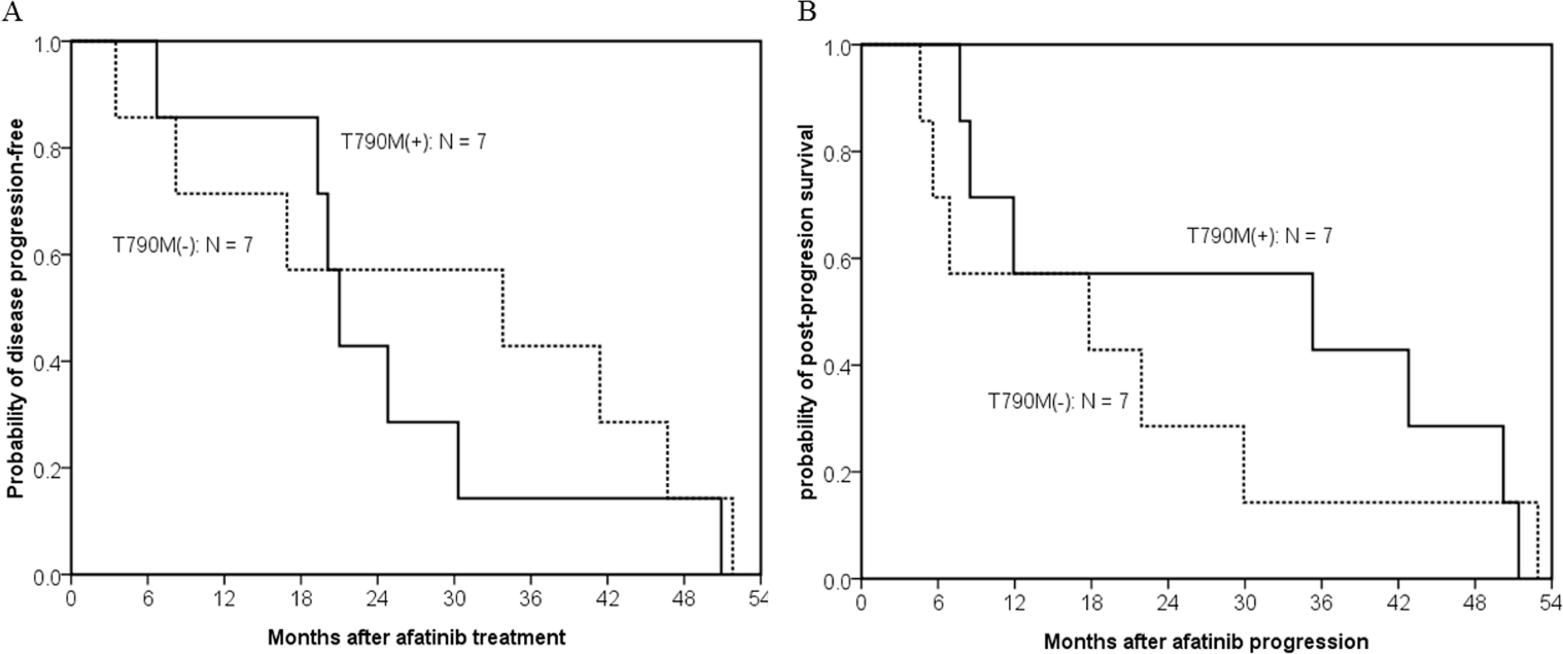
Kaplan–meier curve of post-afatinib-progression survival in 14 first-generation EGFR TKI-naïve patients who acquired resistance to afatinib (**A**) The difference in progression-free survival between patients with (solid line, *n* = 7) and without T790M-mutations (dashed line, *n* = 7) did not reach statistically significant (median, 21.0 months vs. 33.8 months; *p* = 0.648, by the log-rank test). (**B**) Patients with acquired T790M had a longer median post-afatinib-progression survival than patients without acquired T790M mutations, but the difference did not reach statistical significance (median, 35.3 months vs. 17.8 months; *p* = 0.616, by the log-rank test).

To clarify the effect of afatinib, we focused on the 14 first-generation EGFR TKI-naïve patients. PFS were 21.0 months in patients with acquired T790M and 33.8 months in patients without acquired T790M (*p* = 0.648) (Figure 3A). Although patients with acquired T790M had longer median post-afatinib-progression survival (35.3 months) than patients without acquired T790M mutations (17.8 months), the difference did not reach statistical significance (*p* = 0.616) (Figure 3B).

## DISCUSSION

The study showed that approximately half of lung cancer patients with acquired resistance to afatinib had a second *EGFR* T790M mutation. Acquired T790M mutation is still the most common mechanism of acquired resistance to afatinib, a second-generation EGFR TKI. The prevalence of acquired T790M were similar in patients with and without prior exposure to first-generation EGFR TKI. Other acquired resistance mechanisms, including small cell lung cancer or squamous cell transformation, mutation in *PIK3CA, BRAF, HER2, KRAS, NRAS, MEK1, AKT2, LKB1* and *JAK2*, were not detected.

Of the acquired resistance mechanisms of first-generation EGFR TKIs, acquired T790M, was found in approximately 50–60% of *EGFR*-mutant resistance cases [21–23]. The present study showed that acquired T790M in 47.6% of the specimens with acquired resistance to afatinib. This is compatible with prior studies about acquired resistance to first-generation EGFR TKI. In addition, our study showed that the prevalence of T790M in the tissue samples with acquired resistance to afatinib was not different whether patients took first-generation EGFR TKIs before afatinib or not. This may result from that we only enrolled those with partial response or durable stable disease to afatinib. Acquired T790M formation is still the main mechanism of acquired resistance to afatinib.

In addition to T790M, other acquired *EGFR* mutations to first-generation EGFR TKIs, including T854A, D761Y, and L747S, have been reported, but the number of cases were small [14, 24, 25]. The present study did not detect second-site *EGFR* mutations, besides T790M. In addition, our study did not detect acquired C797S mutation which mediates acquired resistance to third-generation EGFR TKI, AZD9291 although afatinib and AZD9291 both acts as an irreversible covalent inhibitor of the EGFR [26, 27]. More cases may be necessary to identify those rare second-site *EGFR* mutations.

Small cell lung cancer transformation has been reported as a mechanism of acquired resistance to EGFR TKI, and it accounts for 3–14% of cases who had acquired resistance to EGFR TKIs [9, 28]. A possible theory may be that both adenocarcinoma and small-cell lung cancer (SCLC) come from a common precursor, alveolar type II cells. When EGFR TKI blocks EGFR signaling related proliferation and differentiation of type II alveolar cells, these cells transform to SCLC if additional key genetic events such as RB1 inactivation occurs [29]. Although our study did not detect cases with SCLC transformation, identification of histological transformation via rebiopsy is still important because results may significantly alters treatment recommendations.

The study did not detect other known mutations in *PIK3CA, BRAF, HER2, KRAS, NRAS, MEK1, AKT2, LKB1 and JAK2*. Ohashi et al. identified acquired *BRAF* mutation in 2 (1%) patients from 195 tumor samples with acquired resistance to erlotinib [16]. Sequist et al. reported that acquired *PIK3CA* mutation was 5% (2 of 37) [9]. The frequencies of acquired *PIK3CA* and *BRAF* mutations were very rare. It may result in undetected acquired *PIK3CA* or *BRAF* mutation in the present study because of our small sample size.

Oxnard et al. showed that patients with T790M had significantly longer PPS [30]. Presence of T790M causes more indolent progression in lung cancer [30, 31]. The present study showed similar results, but the difference did not reach statistical significance. This may result from our small number of patients. More patients are necessary to confirm the result, especially in clinical practice.

Although secondary T790M is still the major mechanism of acquired resistance to afatinib, third-generation EGFR TKI, CO1686 (Rociletinib) and AZD9291, had promising treatment response for patients with resistance to EGFR-TKIs related to T790M mutation [11, 12], with about 60% of response rate and median progression free survival of 9.6–13.1 months [11, 12]. Therefore, finding *EGFR* T790M in tumor specimens after initial therapy with first- and second-generation EGFR TKIs is important to identify patients who may respond favorably to third-generation EGFR TKIs. Rebiopsy after disease progression may be indicated to guide future treatment plans based on different acquired resistance mechanisms.

This study had some limitations. First, the sample size was small although the present study has the largest patient number who had both pre- and post-afatinib tissue samples. However, obtaining tissue specimens for molecular analysis when patients experience disease progression is a persistent problem. Patients may be in extremely poor condition following initial TKI failure, and may not be suitable for rebiopsy. T790M detection from circulating cancer cells or cell-free DNA from blood might change the situation. Second, we did not analyze the amplifications of c-MET, HER2 or FGFR1 by fluorescence *in situ* hybridization because of the small quantity of specimens.

T790M was detected in half of lung adenocarcinoma with acquired resistance to afatinib. T790M is the major mechanism of acquired resistance to afatinib and those with acquired T790M mutations might benefit from the T790M-specific third-generation EGFR TKIs.

## MATERIALS AND METHODS

### Patients and tissue procurement

We retrieved afatinib-prescription records from the pharmacy department of National Taiwan University Hospital (NTUH) from January 2007 to December 2014. We enrolled afatinib-treated patients who had tissue specimens taken after acquiring resistance to afatinib. Patients included in the final analysis had to have both pre-afatinib and post-afatinib tumor specimens available for testing. This study was approved by the NTUH Research Ethics Committee. Some of the pre-afatinib samples were previously examined and reported [32].

The clinical characteristics and medical records were recorded, including demographic information and treatment courses. Patients who had smoked less than 100 cigarettes in their lifetime were categorized as never smokers [33]. Adenocarcinoma histology was confirmed either by pathology reports of the primary or metastatic tumors, or by cell blocks of malignant pleural effusion (MPE) with positive thyroid transcription factor-1 staining by immunocytochemistry [34].

We collected tumor specimens, including frozen tissues of surgical specimens, bronchoscopic or fine needle biopsies, and malignant pleural effusions. Written informed consent to use tissue for molecular analysis was obtained from patients at the time of specimen collection. Tissue sections were examined for adequacy by microscopy with hematoxylin and eosin staining.

### Acquired resistance to afatinib

Categorization as acquired resistance to afatinib was modified from Jackman's criteria [35]. All patients received single-agent treatment with afatinib. They had both tumor harboring a TKI-sensitive *EGFR* mutation and objective treatment response, including partial response or durable stable disease (progression free survival [PFS] ≥ 6 months) to treatment with afatinib. Response Evaluation Criteria in Solid Tumors (RECIST) version 1.1 was adapted to evaluate the objective treatment effect [36]. PFS was defined as days from the date of drug treatment until disease progression or death. Post-afatinib progression survival (PPS) was measured from the date of disease progression under afatinib treatment until the date of death.

First-generation EGFR TKI-naïve group was defined as patients receiving afatinib treatment without prior first-generation EGFR TKI exposure. Patients received prior first-generation EGFR TKIs treatment before taking afatinib were defined as first-generation EGFR TKI-treated group.

### Sequencing of EGFR exons 18–21

Tissue specimens came from lung tumors, metastatic sites and malignant effusion cell blocks. The process of tissue specimen preparation for *EGFR* mutation analysis was as described previously [37, 38]. RNA was extracted from different tissue specimens for gene mutation analysis with Qiamp RNA Mini Kit (Qiagen) according to the manufacturer's protocol. Spectrophotometry was used to quantify extracted RNA.

The cDNA was obtained from the extracted RNA by the Qiagen OneStep reverse transcription polymerase chain reaction (RT-PCR) kit (Qiagen). Exons 18–21 of *EGFR* were amplified. The tyrosine kinase domain of the EGFR coding sequence, exons 18, 19, 20 and 21, were amplified with forward primer (5′- GGA- TCG- GCC- TCT- TCA- TGC-3′) and reverse primer (5′-TAA-AAT-TGA-TTC-CAA-TGC-CAT-CC-3′) by independent polymerase chain reaction (PCR) amplifications. The associated primers and conditions of RT-PCR have been revealed in our prior published reports [32, 39]. Finally, *EGFR* sequences of PCR amplicons were analyzed using ABI PRISM 3100 or 3700 (Applied Biosystems) in both sense and antisense directions.

### Sequencing of PIK3CA, BRAF, HER2, KRAS, NRAS, MEK1, AKT2, LKB1 and JAK2 mutation

Genetic mutations of *PIK3CA, HER2 and BRAF* have been reported as the mechanism of acquired resistance to EGFR TKIs [9, 10]. In addition, other possible gene mutations were also studied in previous report, including, *KRAS, NRAS and MEK1* [28]. After collection of the tissue specimens, we performed a series of genetic mutation analyses, including: *PIK3CA, BRAF, HER2, KRAS, NRAS, MEK1, AKT2, LKB1* and *JAK2*. Genes were amplified by RT-PCR using QIAGEN OneStep RT-PCR kit (Qiagen). The primers of the different genes were listed in the Supplementary Table S1.

RT-PCR conditions were based on manufacturer's protocol. PCR amplicons were sequenced using the same method described for *EGFR* mutation analysis.

### Statistical analysis

Categorical variables were analyzed using Chi-square test. Univariate analysis of patient characteristics was used for comparison between post-afatinib T790M-positive and T790M-negative patients with acquired resistance to afatinib. PFS and PPS were plotted by the Kaplan–Meier method and compared by log-rank test. Two-sided *p*-values less than 0.05 were considered significant. SPSS software (version 17.0 for Windows; SPSS Inc.) was used for all analyses.

## SUPPLEMENTARY MATERIALS TABLE


