# Supplementary material for: The mechanism of acquired resistance to irreversible EGFR tyrosine kinase inhibitor-afatinib in lung adenocarcinoma patients
Source: Oncotarget. 2016 Feb 4;7(11):12404–13. doi: 10.18632/oncotarget.7189 (PMC4914294; doi:10.18632/oncotarget.7189)
Supplement: Supplementary file 1 [file oncotarget-07-12404-s001.pdf]

# The mechanism of acquired resistance to irreversible EGFR tyrosine kinase inhibitor-afatinib in lung adenocarcinoma patients

## Supplementary Materials

### Supplementary Table S1

| Gene | Primer Sequence | |
|---|---|---|
| *EGFR* | Forward | 5′- GGATCGGCCTCTTCATGC-3′ |
| | Reverse | 5′-TAAAATTGATTCCAATGCCATCC -3′ |
| *PIK3CA exon9* | Forward | 5′-TGGTCTGTATCCCGAGAAGC-3′ |
| | Reverse | 5′-GGCCAATCTTTTACCAAGCA-3′ |
| *exon20* | Forward | 5′-ACGTGTGCCATTTGTTTTGA-3′ |
| | Reverse | 5′-GGTCTTTGCCTGCTGAGAGT-3′ |
| *BRAF* | Forward | 5′- TCCAGGACCTCAGCGAGAAAGGA -3′ |
| | Reverse | 5′- TGATGACTTCTGGTGCCATCCACA -3′ |
| *HER2* | Forward | 5′-CCC TCTGACGTCCATCATCT-3′ |
| | Reverse | 5′-TCATCCAACATTTGACCATGA-3′ |
| *KRAS* | Forward | 5′-GGCCTGCTGAAAATGACTGA-3′ |
| | Reverse | 5′-TCTTGCTAAGTCCTGAGCCTGTT-3′ |
| *NRAS* | Forward | 5′-GTCCAAAGCAGAGGCAGTGGAGC-3′ |
| | Reverse | 5′-ACTGTCCTTGTTGGCAAATCACACT-3′ |
| *MEK1* | Forward | 5′- GTCCAAAATGCCCAAGAAGA-3′ |
| | Reverse | 5′- TGTTCAGGAATTCTTCCAGCTT-3′ |
| *AKT2* | Forward | 5′- GAGCGTGTCTTCACAGAGGA-3′ |
| | Reverse | 5′- TAGGAGAACTGGGGGAAGTG-3′ |
| *LKB1* exon1-4 | Forward | 5′- AGTCGGAACACAAGGAAGGAC-3′ |
| | Reverse | 5′- CTGGCTATGCAGGTACTCCAG-3′ |
| exon3-8 | Forward | 5′- GAGAAGCGTTTCCCAGTGTG-3′ |
| | Reverse | 5′- CAAGTACGGCACCACAGTCA-3′ |
| exon7-10 | Forward | 5′- GAAAGGGATGCTTGAGTACGAA-3′ |
| | Reverse | 5′- AACCGGCAGGAAGACTGAG-3′ |
| *JAK2* | Forward | 5′-TCTGATGTACCAACCTCACCA-3′ |
| | Reverse | 5′-AAGGAGGATTTCCTGTCTTCCTG-3′ |
